# Integrin Activation Contributes to Lower Cisplatin Sensitivity in MV3 Melanoma Cells by Inducing the Wnt Signalling Pathway

**DOI:** 10.3390/cancers9090125

**Published:** 2017-09-16

**Authors:** Maria B. R. Piva, Bastian Jakubzig, Gerd Bendas

**Affiliations:** Department of Pharmacy, University of Bonn, An der Immenburg 4, 53121 Bonn, Germany; bethania.piva@uni-bonn.de (M.B.R.P.); bastian.jakubzig@uni-bonn.de (B.J.)

**Keywords:** chemoresistance, cisplatin, integrin, melanoma, PI3K/AKT, wnt pathway

## Abstract

Background: integrins have been associated with the development of chemotherapy resistant tumour cells, mostly those of hematopoietic origin, by mediating the binding to the extracellular matrix. The relevance for solid tumour cells and the underlying mechanisms remain elusive. Methods: using MTT assays, we detected the loss in cisplatin sensitivity of human MV3 melanoma cells upon integrin activation. Underlying cellular pathways were evaluated by flow cytometry. A crosstalk between integrin activation and the canonical wnt signalling pathway was tested by measuring β-catenin activity. Results: MV3 cells display a higher resistance against cisplatin cytotoxicity when cellular integrins were activated by manganese or collagen. Proteome profiler array showed a deregulation of the integrin expression pattern by cisplatin. Integrin activation by manganese induces the phosphorylation of PI3K/AKT. The inhibition of PI3K using BEZ235 strongly increases cell sensitivity to cisplatin, blocking manganese and collagen effects. PI3K/AKT activates wnt signalling by blocking Gsk3-β, which was confirmed by β-catenin up-regulation and nuclear localization. Integrins did not affect E-cadherin expression levels, thus endothelial to mesenchymal transition (EMT) can be excluded. Conclusion: This is the first report on an integrin/wnt signalling activation axis addressing the consequences for chemotherapy sensitiveness of melanoma cells, which thus offers novel therapeutic targets for approaches to interfere with chemoresistance.

## 1. Introduction

Integrins are ubiquitous cell membrane proteins that are involved in cell adhesion and signalling. They are known to be the main receptors for the extracellular matrix (ECM), associating cells with collagen, fibronectin, and laminin among other substances [1,2,3]. By binding to the ECM, or cell surface receptors, integrins regulate not only simple physical cell attachment, but also activate specific signalling pathways, which enhance tumour cell migration, invasion, proliferation, and survival [1,2,3,4,5]. Therefore, integrins have been linked to cancer progression. The malignancy of melanoma cells, for example, is often associated with their integrin expression pattern. The expression of integrin α5β1 and αvβ3 have been indicated to result in a poor melanoma prognosis, increased cell invasion, and metastasis [6,7]. Moreover, the inhibition of these integrins has been shown to decrease melanoma metastasis and angiogenesis in vivo [8,9]. Nonetheless, the underlying mechanisms through which integrins impact malignancy are elusive and still far from being understood.

Tumour cells make use of a number of potential molecular mechanisms to adapt to chemotherapeutic treatment and circumvent cytotoxicity. Despite many innovations in cancer pharmacotherapy, drug resistance still represents one of the most important obstacles to clinical cancer patient treatment [4,5,10]. Resistance develops as tumour cells seek refuge in protective niches, where the microenvironment provides tumour cells the opportunity to adapt to chemotherapeutical stress through genetic modifications. Finally, this results in a reduced sensitivity to treatment by an acquired drug resistant phenotype [4,10].

The role of the microenvironment in the initial protection of tumour cells is mediated by the interaction between tumour surface receptors and soluble factors or components of the ECM. This phenomenon is known as “cell-adhesion-mediated drug resistance” (CAM-DR). Integrin expression has been associated with this phenomenon due to its role in the interaction with ECM molecules [4,5,10]. It has been shown that integrins trigger signal cascades that help cancer cells survive chemotherapeutic treatments [1,4,5,10]. One example is the phosphatidylinositol 3-kinase (PI3K)/AKT pathway. This pathway can be activated as a result of integrin clustering via focal adhesion kinase (FAK) [4,11,12]. FAK dimerization and auto-phosphorylation at Y397 leads to Src protein kinases recruitment, phosphorylation of FAK at Y576, and Y577 generating an active FAK-Src complex. This complex activates PI3K/AKT signalling through phosphatidylinositol-4,5-diphosphate [PtdIns(4,5)P2] (PIP2) [13,14,15,16]. In head and neck cancer, the blockade of integrin-FAK signalling by integrin β1 antibodies sensitized cells to radiotherapy and delayed tumour growth in vivo [17]. Moreover, it has been described that integrin activation in ovarian cancer induces resistance to paclitaxel and prevents cell-death in an integrin-FAK-AKT-dependent manner [18,19]. On the other hand, FAK inhibitors have shown to enhance cancer cell sensitivity to cytotoxic drugs [19,20].

PI3K, and its downstream effector AKT, are frequently inappropriately activated and associated with malignancy in several different tumours, including melanoma [4,12,21,22,23]. Once activated, AKT can phosphorylate a wide variety of substrates involved in various cellular processes, including cell growth, proliferation, survival, and metabolism, all of which are crucial for the establishment and the maintenance of the tumorigenic phenotype [21].

One of those substrates is the glycogen synthase kinase 3β (Gsk3-β), a typical serine/threonine kinase [24]. Gsk3-β has a significant role in the wnt signalling pathway, which is directly associated with the pro-tumorigenic and metastatic development in several cancer types. Wnt signalling pathway is often implicated in stem cell control. Abnormal wnt signalling is especially involved in cancer, as it mediates stem cell-like renewal and proliferation, which predisposes cells to tumorigenesis [25]. It is known that wnt signalling is important in the development of melanocytes in melanoma cells [26]. Fully developed melanoma is capable of resisting certain anti-tumorigenic processes through wnt signalling [27]. For example, the cytotoxic T-lymphocyte antigen-4 (CTLA-4), which is known to inhibit immune response, is up-regulated in melanoma cells through wnt signalling [28]. However, a potential link between wnt signalling and reduced sensitivity to chemotherapy in melanoma has not yet been established.

Wnt signalling is divided into non-canonical and canonical pathways. The latter is also known as wnt/β-catenin pathway, which is activated by wnt ligands binding to the Frizzled-receptor (Fzd). Fzd is a transmembrane G protein-coupled receptor interacting with low-density lipoprotein receptor LPR5/6 at the cell surface. In the off-state, β-catenin is degraded by a destruction complex. This complex includes Gsk3-β, which phosphorylates β-catenin finally leading to its ubiquitination and degradation in the proteasome. If a wnt ligand is attached to Fzd, dishevelled (Dvl) recruits axin and Gsk3-β to the cell membrane, leading to the disassembly of the destruction complex. This then allows for stabilized β-catenin to accumulate in the cytoplasm before translocating into the nucleus, where it binds to transcription factors of the T-cell factor and the lymphoid enhancer factor (TCF/LEF) family. Ultimately, wnt targeted genes are activated, which results into cell proliferation and survival. Key molecules, such as cyclin D1 (CCND1) and survivin (BIRC5), whose deregulation is closely related to cancer, were found among the more than 100 targeted genes activated by wnt signalling [29,30,31].

Beside the above mentioned wnt ligand mediated activation, wnt signalling is also influenced by the localisation of E-cadherin. E-cadherin is part of a family of transmembrane proteins mediating cell-cell adhesion. It forms a complex with β-catenin that directs downstream β-catenin activation [32]. E-cadherin function is often associated with cell transition from epithelial to a mesenchymal phenotype (EMT) [33]. In fact, data show that wnt signalling can be a consequence of E-cadherin loss at the cells tight-junctions, leading to intracellular enrichment of β-catenin. In ovarian cancer cells, Burkhalter et al. demonstrated that the delocalisation of E-cadherin from the tight junctions of cell-cell contact through integrin clustering led to increased β-catenin levels inside the nucleus, and the transcription of wnt targeted genes [34].

Consequently, data suggest that integrins can trigger the wnt pathway by different modes of action: via the PI3K/AKT signalling axis, or affecting cell contacts, thus changing the E-cadherin functionality. Whether this relation contributes to an attenuated sensitivity towards chemotherapeutics has not yet been investigated. On the other hand, integrins are known to play a certain role in CAM-DR and reduce tumour cell chemosensitivity.

In this work we were able to show that activation of integrins reduces the sensitivity towards cisplatin cytotoxicity induced by the PI3K/AKT pathway, in human melanoma MV3 cells. Reduced sensitivity is directly related to an up-regulation of wnt activity, independent of a deregulation of E-cadherin. Our data support a model of integrin-driven CAM-DR as an initial step for acquiring chemoresistance, and suggest integrins and their pathways as targets to interfere with chemosensitivity.

## 2. Results

### 2.1. Integrin Activation in MV3 Melanoma Leads to Decreased Sensitivity against Cisplatin Cytotoxicity

In order to investigate whether integrin activation has had an impact on MV3 human melanoma cells sensitivity to cisplatin, cells were either treated with manganese, which specifically activates integrins by inducing clustering and high affinity binding state, or grown on collagen type-I coated surfaces. Alternatively, a combination of both treatments was also used. Cell sensitivity to cisplatin was evaluated via MTT (3-(4,5-dimethylthiazol-2-yl)-2,5-diphenyltetrazolium bromide) cytotoxicity assay after 72 h.

**Figure 1:**
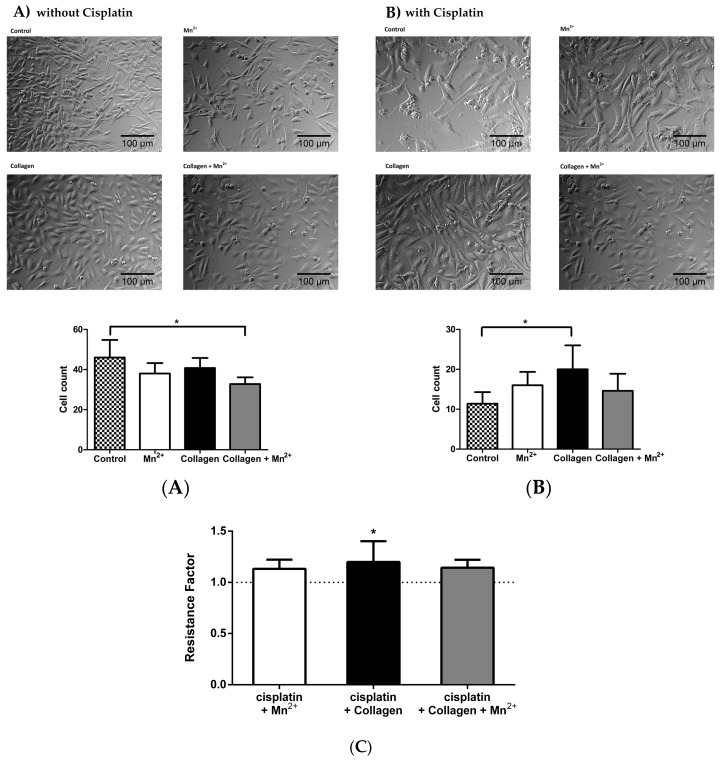
(**A**) Exemplary microscopy images of MV3 cells, cultured for 72 h, under the indicated conditions (control, collagen or/and manganese) without cisplatin. (**B)** Exemplary microscopic pictures of MV3 cells, cultured for 72 h, under the same indicated conditions, but in the presence of cisplatin (7.5 μM). Pictures were taken at five different growth spots (for all treatments) and cells were then counted in a predefined area. The graph displays the mean of cells counted for the different spots (*n* = 5), which were then analysed by one-way ANOVA. Asterisks indicate statistical significance: * *p* < 0.05; ** *p* < 0.01; *** *p* < 0.001. (**C**) Resistance factors (ratio between IC_50_ of treated vs. control) of MV3 cells as indicator for sensitivity to cisplatin cytotoxicity.

In order to exclude a potential impact of the integrin activating treatments on the cell proliferation rate, cell counts were observed microscopically, after 72 h, in the presence or absence of cisplatin. As indicated in Figure 1A, neither manganese nor collagen or the combined treatment increased the cell proliferation rate. On the contrary, cell growth and density seemed to be slightly reduced, when compared to the control cells. In strict contrast, when cells were exposed to a cisplatin concentration of 7.5 μM, for 72 h, the integrin-activated cells displayed a clearly higher proliferation capacity than the untreated control cells (Figure 1B). Consequently, any changes in IC_50_ values presented in Table 1 do not originate from an integrin-activated accelerated cell proliferation rate, but it appears to be the first indication for a lower sensitivity to cisplatin cytotoxicity in the integrin activated cells, when compared to the respective controls. As a common expression of lower chemosensitivity, the ratio between IC_50_ of treated vs. control cells, referred to as resistance factor (RF) is illustrated in Figure 1C. In the case of collagen treated cells there was a statistically significant difference between the RF and control cells. On the other hand, manganese and the combination of both treatments induced a consistent, but not statistically significant, increase of the RF. These findings point to a possible relationship between integrin activation and a loss in cisplatin sensitivity, which we further examined.

**Figure 2:**
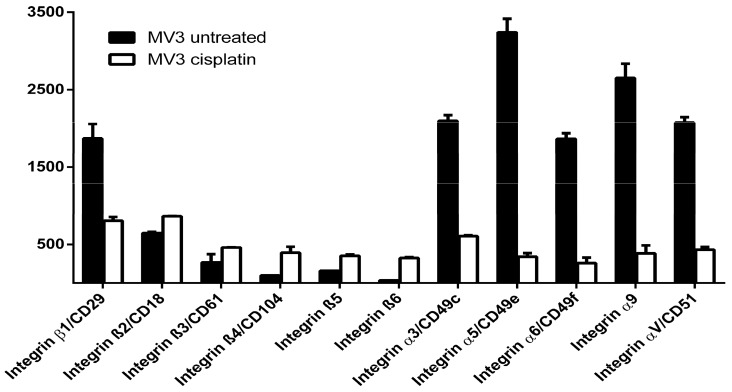
Integrin expression pattern of MV3 melanoma cells and the impact of cisplatin treatment. Cells were incubated for 72 h in the presence of cisplatin (IC_50_) (white) and then washed twice with PBS (phosphate buffered saline) to exclude dead cells before being submitted to proteome profiler array compared to untreated cells (black). The array revealed a strong decrease in a great variety of proteins (data not shown) and a shift in integrin distribution among its subunits representing one of only a few up regulations in proteins, under cisplatin treatment. All α-chains of integrins investigated were strongly down-regulated, while the ratio of β-chain containing integrins was, with exception of β1, up-regulated. β1 and α3 are known to be receptors binding to collagen. The decrease of collagen binding integrins and shift to other integrin subtypes serves as first hallmark for the observed differences in integrin activation. Measurements were conducted in duplicates (*n* = 2). The individual differences between the two measurements were mostly below 10%.

The data above indicate that the integrin activity affects the cisplatin sensitivity of MV3 cells. To first consider the potential integrin/cisplatin functional axis at a protein expression level, we checked whether cisplatin exerts a specific impact on cellular integrins. A protein profiler array was performed in order to detect the integrin expression pattern of untreated and cisplatin treated MV3 cells. Representative data concerning the integrins are presented in Figure 2. Under the influence of cisplatin at a concentration of 7.5 μM, a dramatic decrease in nearly all of the profiled proteins was evident (data not shown). Interestingly, the only proteins that were upregulated by the cisplatin treatment belong to the integrin fraction. Thus, the overall integrin expression pattern undergoes a shift in the sub-type expressions. On the one hand, there is a down-regulation of several of those integrin subunits, which are important for collagen binding, such as β1 and α3. On the other hand, integrin subunits β4, β5 and β6, which are barely detectable in untreated cells, were more strongly expressed under cisplatin stress. Cisplatin clearly alters the integrin expression pattern of MV3 cells, possibly indicating a direct link between the increased levels of integrin subtypes and the decreased cell sensitivity in the presence of cisplatin.

### 2.2. Resistance of MV3 Cells Is Mediated through the PI3K/AKT pathway

To further elucidate the path through which integrin activation reduces the cellular sensitivity to cisplatin, we investigated the potential downstream key effectors of integrin signalling: PI3K, and its direct target of phosphorylation, AKT. Therefore, the cells were treated with a cisplatin concentration of 7.5 μM for 72 h, in accordance to the treatment regimens as used for the MTT assays and subjected to a flow cytometric analysis.

**Figure 3:**
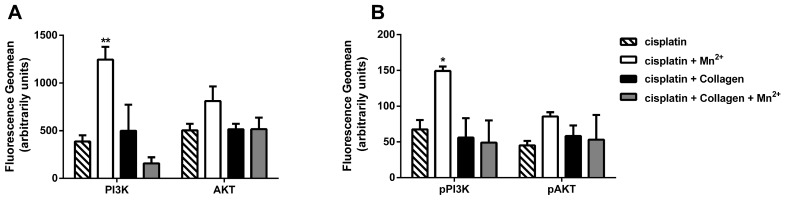
Protein levels of PI3K and AKT assessed via FACS (fluorescence activated cell sorting) after 72 h of cisplatin incubation. (**A**) Total levels of PI3K and AKT. Data indicate a significant increase in PI3K, and slight up-regulation in AKT, only in the manganese treated cells. (**B**) Phosphorylated fractions of PI3K and AKT. Results show a significant increase in pPI3K in the manganese-treated cells and gain in pAKT. Presenting geomean of at least *n* = 3 (SEM), asterisks indicate statistical significance compared to only cisplatin treated cells: * *p* < 0.05; ** *p* < 0.01; *** *p* < 0.001.

Interestingly, only in case of the manganese, which is a known integrin-specific activator, a significant increase in PI3K and a distinct gain in AKT are evident (Figure 3A). Furthermore, this is accompanied by consistently increased levels of the phosphorylated form of these enzymes (Figure 3B). However, these upregulations were not evident under collagen treatment, or the combined treatment. Although the PI3K/AKT signalling is involved in various cell processes, and can be triggered by several different cell receptors, the fact that PI3K/AKT increase was present only under manganese stimulation indicates an integrin related phenomenon. It seems difficult to understand why only under manganese treatment the activation of the PI3K/AKT axis in the presence of cisplatin is clearly detectable. This could be related to the change in the integrin expression pattern, as shown in Figure 2, and therefore a lower susceptibility of the changed integrin subunits for collagen binding, or due to other cell receptors and pathways, which are still to be investigated.

Although we cannot take the strong up-regulation of PI3K and phosphorylated PI3K in the manganese treated cells as an ultimate indicator for integrin signalling, we tested the association of such findings with cisplatin sensitivity. We blocked PI3K with the PI3K inhibitor BEZ235 and investigate its impact on cisplatin sensitivity. BEZ235 binds reversibly and competitively to the ATP-binding cleft of PI3K and mTOR, working as a potent and selective inhibitor of PI3K pathway. BEZ235 presents a very low affinity to other down- or upstream effectors, such as FAK, and PDK1, but has been shown to interfere in 50% of AKT phosphorylation in a reversible manner [35]. Cells were treated with a concentration of 100 nM of BEZ235, which possesses low cell toxicity (Supplementary Figure S1). Cells were exposed to BEZ235 for a period of 6 h, before cisplatin was added; subsequently, they were incubated with both compounds for 72 h.

**Figure 4:**
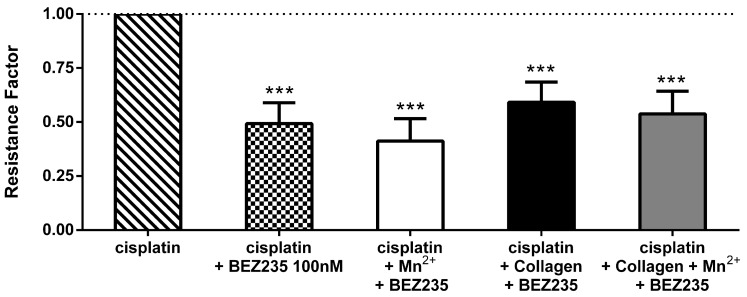
Change in cisplatin resistance factors under PI3K inhibition. Cells (10^4^ cells/well) were incubated with integrin activators concomitantly with PI3K inhibitor BEZ235 (100 nM) for 6 h, before the addition of cisplatin. BEZ235 is depicted alone or with additional integrin activating stimuli: treatment with 1 mM manganese or collagen coating. After 72 h, MTT assay was performed revealing a strong increase in sensitivity in all cells treated with the BEZ235. This highlights the relevance of PI3K signalling for cells to survive cisplatin treatment and suggests an important role of integrins in cell survival against chemotherapeutic treatment. Measurements were conducted in triplicates (at least *n* = 3). Asterisks indicate statistical significance: * *p* < 0.05; ** *p* < 0.01; *** *p* < 0.001.

Our results show (Table 2) that the inhibition of PI3K by BEZ235 dramatically increases the sensitivity, indicated by a more than bisected IC_50_. Interestingly, in the presence of BEZ235, the integrin-activated cells neither differ from this sensitization, nor display remarkable differences among each other and possess comparable IC_50_. That is further illustrated by the similarity in the RFs (Figure 4). This might indicate that the blockade of PI3K interferes with an essential key point of integrin activating stimuli that mediate the resistance formation. However, these findings are partly contradicted by PI3K upregulation solely by manganese, which points to other possible pathways, which could not be evaluated at this point. Nevertheless, the data confirm the strong impact of PI3K on cisplatin sensitisation of the cells.

### 2.3. Wnt Signalling as Keystone between Integrins and Chemoresistance in MV3 Cells

**Figure 5:**
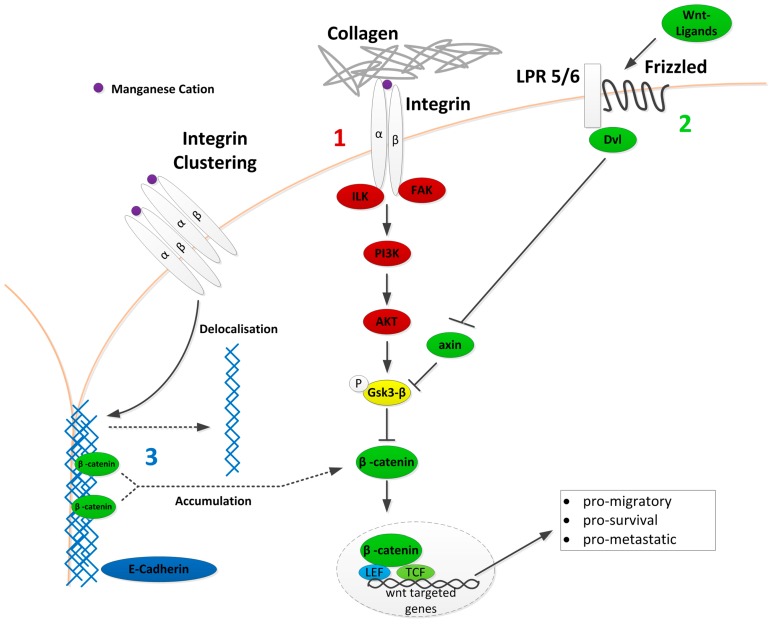
Crosslinks between integrin and wnt signalling. A schematic overview upon important steps of wnt signalling (green) and PI3K/AKT signalling (red) with Gsk3-β (yellow) as centrepiece connecting both pathways. The blue section highlights the involvement of E-cadherin delocalisation through integrin activation in β-catenin accumulation in the nucleus. Canonical wnt signalling begins with the connection between LPR 5/6 and Fzd leading to the recruitment of Dvl and deactivation of axin, which is no longer able to form the destruction complex, leading to Gsk3-β destabilisation. Active wnt signalling ultimately leads to the transcription of wnt-targeted genes that are known to transcribe into pro-migratory, pro-survival, and pro-metastatic factors. After integrin activation, a signalling cascade involving a multitude of proteins is activated, two of which are PI3K and AKT, whose main phosphorylation target is Gsk3-β, therefore, connecting the two pathways. Moreover, E-cadherin delocalisation, through integrin activation, has been shown to release β-catenin into the cytoplasm, where it can act as an effector on the wnt-pathway.

Taking the relation between integrin activation pathway and cell resistance, we aimed to elucidate the underlying mechanisms involved in this process. Several mechanisms have been described to be involved in cisplatin resistance; one of these is the wnt signalling pathway, which could be affected by integrins, as illustrated in Figure 5. This schematic depicts our hypothesis of a possible connection between integrin and wnt signalling pathway, as well as E-cadherin interaction in this context. 

Integrin signalling through the PI3K/AKT axis, indicated in red (1), intersects with the canonical wnt signalling, displayed in green (2), through the key molecule Gsk3-β (yellow) that knits both of the pathways together. Gsk3-β serves as phosphorylation target for AKT. Normally, axin binds to Gsk3-β stabilising each other to form a destruction complex that accelerates β-catenin phosphorylation and degradation. An additional mechanism, displayed in blue, indicates the impact of integrins on E-cadherin localisation in the tight junctions. As shown by Burkhalter et al., integrin clustering causes E-cadherin delocalisation that leads to an intracellular increase in β-catenin and activation of wnt signalling [34].

**Figure 6:**
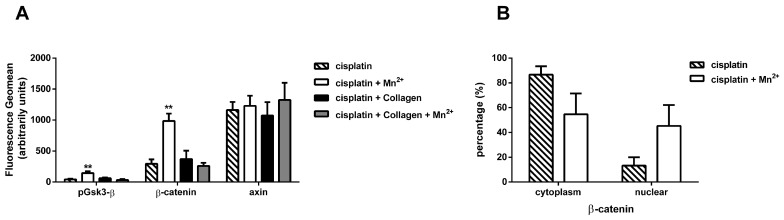
Wnt signalling. Protein levels were accessed by FACS after 72 h of incubation with cisplatin (IC_50_). MV3 cells were treated as indicated above. Cells were collected after 72 h of cisplatin incubation and analysed. (**A**) Total protein level of wnt signalling related proteins. Data show a significant increase in pGsk3-β as well as β-catenin indicating active wnt signalling. Note that there is no apparent deregulation of axin excluding the possibility of canonical activation through frizzled. Presenting geomean of at least *n* = 3 (SEM); asterisks indicate statistical significance compared to cells treated exclusively with cisplatin: * *p* < 0.05; ** *p* < 0.01; *** *p* < 0.001. (**B**) β-catenin localisation was accessed by FACS only for cisplatin treated cells and manganese-cisplatin combination, based on the results of Figure 6A. Total and partial cytoplasmic fraction of β-catenin was measured, from which nuclear fraction was calculated. Data demonstrate strong translocation of β-catenin inside the nucleus, when integrins are activated by manganese, showing the geomean of *n* = 3 (SEM).

We demonstrated above that integrin-specific activation through manganese increased both PI3K and AKT expression, and phosphorylation. In order to show the impact of these proteins in the wnt signalling pathway, we first evaluated the levels of the two key molecules connecting these pathways, Gsk3-β, and its target β-catenin by fluorescence flow cytometry (Figure 6A).

We observed a significant increase in the amount of phosphorylated Gsk3-β in the manganese treated cells. Gsk3-β phosphorylation at serine 9 by AKT indicates a Gsk3-β deactivation leading to enhanced wnt signalling. Consequently, the levels of β-catenin are significantly higher under manganese treatment as a direct result of the deactivation of Gsk3-β.

To exclude canonical wnt activation through axin, and to demonstrate that the effect observed was indeed a result of upstream integrin stimulation, we investigated the cellular expression of axin under cisplatin and the different treatments. Interestingly, there was no change in the axin expression levels under any of the treatments (Figure 6A). This suggests that wnt signalling is not regulated through the normal frizzled-axin axis, as depicted in red in Figure 5, but it is aberrantly activated by integrin-signalling through inhibition of Gsk3-β, as a result of cisplatin treatment. In line with these findings, we could observe that under manganese treatment, the nuclear fraction of β-catenin is strongly elevated (Figure 6B). 

### 2.4. Integrin Activation and Clustering Does Not Influence E-Cadherin Expression

**Figure 7:**
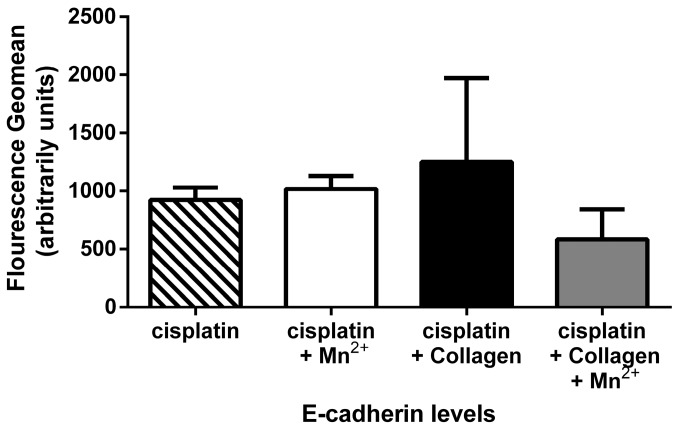
E-cadherin expression. E-cadherin protein levels were accessed by FACS after 72 h of incubation with cisplatin (IC_50_). MV3 cells were grown in cell culture flasks either coated with collagen type I or uncoated and, depending on the treatment, incubated with manganese enriched cell culture medium, for 5 min, prior to the addition of cisplatin. Results show no detectable deregulation in E-cadherin levels, considering the geomean of at least *n* = 2 (SEM).

Integrin activation and clustering has been shown to have an impact on the localisation of E-cadherin in ovarian carcinomas [34], which consequently affects β-catenin. For this reason, we also considered E-cadherin as a potential target of integrin activity. E-cadherin, usually located at the cells tight-junctions and also found within adherens-junctions, connecting cells to each other enabling them to form tissue. It is associated with β-catenin. If E-cadherin is delocalized from the cell-junction to other cell compartments, this delocalisation is accompanied by a release of β-catenin, which was previously bound to E-cadherin. This leads to an enrichment of β-catenin in the cytoplasm. For further examination, we detected the E-cadherin levels within the MV3 cells by flow cytometry. As indicated in Figure 7, E-cadherin levels were not affected by integrin activation with manganese or/and collagen, before the addition of cisplatin, which was also confirmed by western blot results (Supplementary Figure S2). Consequently, it is safe to assume that wnt signalling is activated via the PI3K/AKT signalling likely triggered by manganese-activated integrins. E-cadherin is also associated with the process of endothelial to mesenchymal transition (EMT). Another protein typically de-regulated in this process is vimentin. To explore whether EMT is induced by integrin activation, and thus affect the cisplatin sensitivity, we detected vimentin through western blot (Supplementary Figure S2). Since no changes in vimentin and E-cadherin were observed, EMT involvement in this scenario appears unlikely.

### 2.5. Wnt as Mechanism of Integrin Mediated Loss of Sensitivity

For further insight, we investigated whether the up-regulation of β-catenin induced by manganese results in an activation of the wnt signalling pathway. Therefore, we applied the TOPflash assay. For this purpose, MV3 cells were transfected with a TCF/LEF reporter plasmid containing the TCF/LEF promotor region and the firefly luciferase gene. If wnt signalling is active at the transcription level, luminescence readout is generated after substrate addition. Unfortunately, the already indicated activity changes in Gsk-3β and β-catenin could not be replicated at the transcriptional level using this assay (Supplementary Figure S3). The manganese treated cells tend to display a slightly higher transcriptional activity, but this increase was not significant. However, we failed to detect any changes in the activity of wnt in the collagen and the combined treatment groups, which reflects the context of our previous findings.

## 3. Discussion

In the present work, we provide evidence that integrin activation of melanoma cells has a direct functional impact on attenuating the sensitivity of such cells to cisplatin cytotoxicity. Our data support a model of an integrin-driven PI3K/AKT signalling, which intersects, and thus activates, the canonical wnt pathway by phosphorylation and deactivation of Gsk3-β. This appears to be an integrin-wnt activation pathway, independent of E-cadherin deregulation, as the triggering of wnt activity by the latter was excluded. Consequently, these data shed new light onto integrins, known contributors for tumour cell malignancy, relating them to chemoresistance and the wnt pathway. This growing insight introduces a novel and promising topic, which has very recently also been explored by Kolev et al. [36].

Tumour cells’ resistance against chemotherapeutic treatment remains the major obstacle in the clinical treatment of cancer patients. Several molecular mechanisms of tumour cell resistance have been described, including the up-regulation of efflux transporters [37], boosted DNA repair [38,39], and several others, which are elusive and vary depending on the tumour entities. However, all of these modifications result into genetic adaptations of cells to chemotherapy. For example, we were recently able to show that cisplatin resistant A2780 ovarian cancer cells differ in more than 1500 genes from their cisplatin-sensitive counterparts [40]. As an initial step, and as a functional prerequisite for the slow adaptation, tumour cells have to be temporally protected to survive cytotoxic stress. It is known that the interaction with matrix components contributes to this survival, which is clinically evident from the pathological phenomenon of minimal residual disease (MRD) [10]. In this context, CAM-DR contributes to further disease development. It is one of the building blocks of the chemoresistant phenotype. Cell membrane proteins interact with ECM components rapidly inducing signalling events that lead to survival. Naturally, integrins as major cell receptors have been strongly associated to this phenomenon [4].

Our data support this hypothesis of integrin contribution, and point to an intracellular mechanism to explain the increase in resistance. Canonical wnt signalling is a pathway of major importance in various tumour entities driving tumorigenicity on various functional axes, and therefore, emerges as an attractive target for therapeutic approaches [25]. However, our attempts to intensify chemoresistance via integrin activation only slightly affected cisplatin toxicity by a 10 to 20% increase in IC_50._ Nevertheless, we believe that this is an essential step to tackle chemotherapy resistant tumours. In light of the non-genetic nature of this rapid adaptation, these data appear realistic when compared to resistance factors of genetically modified resistant cells, which typically range from 2 to 10-fold. Moreover, our studies have been performed with a classical 2D-cell culture model, which can hardly simulate the ECM contact formation in the tumour tissue. Therefore, further activities will be directed to transfer the cell to a 3D-cell culture system in the future.

Although the data using manganese as an integrin-specific activator seem to be explained by downstream signalling through PI3K/AKT pathway, thus affecting wnt signalling; data from integrin activation by collagen binding and the combination of both treatments appear controversial. We speculate that another pathway must be responsible for inducing the lower sensitivity, as collagen induced a greater loss of sensitivity than manganese, but clearly did not follow PI3K/AKT pathway. The fact that the combination of treatments did not present the synergistic effect that we expected, but it actually seemed to antagonize itself, also supports this hypothesis. 

Referring to the PI3K/AKT/Gsk3-β axis defined here, it must also be considered that other pathways use Gsk3-β as mediator for their signalling. For example, Gsk3-β is involved in the process of EMT not only via the wnt regulation, but also by regulation of the zinc-finger transcriptional repressor SNAIL and hedgehog [41]. Furthermore, protein kinase A (PKA), protein kinase C, p70 S6 kinase, and other kinases do also phosphorylate Gsk3-β at Ser9 [42]. This could also strengthen the deactivation of Gsk3-β and therefore contribute as potential targets of pathway crosstalk within cancer cells. 

This indicates that integrin activation leading to increased chemoresistance is a complex process, which is regulated by different signalling pathways and probably depends on the integrin subunits and the mode of activation. Thus, it must be considered that collagen is only bound by specific integrin subunits and can also bind to other cell surface proteins. In contrast, manganese is known to be a specific ligand to integrins. Manganese changes the integrin conformation, it is essential in heterodimer formation and also induces integrin clustering [43,44]. We believe that the changes in the integrin pattern triggered by cisplatin influences the cell signalling, which leads to a reduced sensitivity to treatment. 

EMT, which is also held responsible for increased tumour malignancy in the process of metastatic spread, appears unlikely to be involved in this scenario, considering the unchanged total expression of E-cadherin, as shown by the western blot and flow cytometry.

In summary, the potential link between integrin activation and loss in tumour cell sensitivity for cytotoxic drugs sheds a new light on integrins in cancer, as well as offers novel therapeutic targets for promising approaches. Understanding the role of integrins in the onset of resistance formation could be a target to block resistance formation, before genetic adaptation occurs. Several questions remain to be answered. Therefore, we will continue our search for these explanations and for a model that is applicable to other tumour entities.

## 4. Materials and Methods

### 4.1. Cell Culture and Reagents

The MV3 amelanotic melanoma cell line was established, as described by van Miujen [45], and has been thoroughly characterized. Cells were kept in RPMI 1640 medium supplemented with penicillin (10 IU/mL), streptomycin (100 μg/mL), and 10% FBS, in humidified atmosphere, at 37 °C containing 5% CO_2_. Cells were detached using a solution of EDTA (0.2 g/L EDTA × 4 Na), for 3 min, at 37 °C. All reagents are from Thermo Fisher Scientific (Waltham, MA, USA).

Cisplatin (CDDP) (Sigma-Aldrich Chemie GmbH, Taufkirchen, Germany) has been suggested as a potential therapy for chemotherapy-resistant melanoma. Cells were treated for 72 h with different doses of CDDP (1 × 10^−3.5^ mol/L–1 × 10^−7.5^ mol/L), diluted in PBS (phosphate buffered saline), under different conditions, in order to simulate physiological events that may be related to CAM-DR.

Collagen, which has been shown to bind to integrins and stimulate cisplatin chemoresistance in several cancer types [46,47,48] was used to simulated the CAM-DR processes. 96-well plates were coated with collagen (Roche Diagnostics GmbH, Mannheim, Germany) at a density of 10 μg/cm^2^, according to the manufacturers protocol. For flow cytometry and western blot experiments, collagen coated cell flasks were used (Sarstedt AG & Co., Nümbrecht, Germany)Manganese (Mn^+2^) solution (1 mM) was added to the cells, for 5 min, prior to cisplatin treatment, in order to specifically activate integrins [46,49,50,51]. BEZ235 (Dactolisib), is an imidazole[4,5-c]quinolone derivative dual pan-PI3K/mTOR inhibitor (Selleck Chemicals, Huston, TX, USA), and was used to block the PI3K pathway. Cells were treated with the non-cytotoxic concentration of 100 nM of BEZ235 (Supplementary Figure S1), for 6 h, and then incubated concomitantly with different concentrations of cisplatin, for 72 h, until the MTT assay was conducted.

### 4.2. Microscopy

Cells were cultured, as described in Section 4.1., in T25 cell culture flasks. Samples consisted of control cells and cells treated with collagen, manganese, or both components combined. These cells were either incubated with or without cisplatin (7.5 μM). Pictures were taken at 20× at five different growth spots (equal among all treatments) and cells were then counted in a predefined area. Axiovert 200 inverted microscope (Carl Zeiss GmbH, Jena, Germany) was used.

### 4.3. MTT Assay

The MTT assay was conducted using 3-(4,5-dimethylthiazol-2-yl)-2,5-diphenyltetrazolium bromide (BioChemica, Applichem GmbH, Darmstadt, Germany) to measure cytotoxic activities of CDDP as described in [52]. Cells were seeded at a density of 5 × 10^3^ cells/well in 96-well plates (Sarstedt AG & Co.). For collagen treatment, plates were previously coated with collagen, as described above. Plates were analysed under a spectrophotometer at 570 nm, with background subtraction at 690 nm, using a plate reader (Thermomultiscan EX, Thermo, Schwerte, Germany).

### 4.4. Flow Cytometry

Cells were detached from the cell culture flasks with 0.02% EDTA, and flow cytometry analysis was carried out as described in [53]. Cells were incubated for 2 h with rabbit anti-β-catenin, anti-pAKT (Thr308), anti-PI3K-p85α, anti-axin (GeneTex Inc., Irvine, CA, USA), anti-E-cadherin, goat anti-pGsk3-β (Ser9), anti-AKT, and anti-pPI3K p85α (Tyr 508). After that, the cells were incubated for 2 h with the appropriate secondary antibody: donkey anti-rabbit IgG Alexa Fluor 405-conjugated (Abcam, Cambridge, UK), donkey anti-goat IgG-FITC-conjugated. All antibodies were diluted in PBS with 1% albumin, and all non-specified antibodies were purchased from Santa Cruz Biotechnology (Dallas, TX, USA). Data were obtained using a Guava^®^ easyCyte Flow Cytometer (Merck Millipore, Billerica, MA, USA).

### 4.5. Proteome Profiler™ Antibody Array

A Proteome Profiler™ Antibody Array kit (R&D Systems GmbH, Wiesbaden-Nordenstadt, Germany) was performed to screen MV3 cells for certain membrane proteins and the impact of cisplatin treatment thereof. Concisely explained, MV3 melanoma cells were washed twice with PBS to exclude dead cells, before the cell lysis buffer provided in the kit was added; cell lysates were prepared following the manufacturer’s instructions. Then, Pierce™ BCA Protein Assay Kit (LifeTechnologies, Thermo Fisher Scientific Inc, Waltham, MA, USA) was used to quantify total protein. Then, the assay was performed following the manufacturer’s instructions. The proteins present in the antibody arrays were quantified via chemiluminescence after membrane treatment with Clarity Western ECL substrate chemiluminescence kit (BioRad Lab GmbH, Munich, Germany). Membranes were photographed and quantified using ChemiDoc XRS+ imaging acquiring system (BioRad), and Image Lab software (BioRad).

### 4.6. Western Blot

Cells were washed twice to exclude dead cells. Then, cells were lysed using extraction buffer (Life Technologies, Carlsbad, CA, USA), supplemented with phenylmethanesulfonylfluoride (0.1 mM PMSF) (Life Technologies, Carlsbad, CA, USA) and a protease inhibitor cocktail (1 μg/mL aprotinin, 1 μg/mL leupeptin) (Life Technologies, Carlsbad, CA, USA), according to manufacturer’s instructions. Cells were then centrifuged and supernatant was collected and submitted to protein quantification by Pierce™ BCA Protein Assay Kit (Thermo Fisher Scientific Inc, Waltham, MA USA). SDS-Page and Western Blot were performed, as described in [54]. Membranes were incubated with rabbit anti- E-cadherin (Santa Cruz Biotechnology, Dallas, TX, USA), mouse anti-GAPDH (GeneTex, Irvine, CA, USA), and goat anti-rabbit and donkey anti-mouse IgG HRP-conjugated (Santa Cruz Biotechnology, Dallas, TX, USA) diluted in 1% BSA solution. Western Blot was quantified via chemiluminescence using Clarity Western ECL substrate chemiluminescence kit (BioRad). Membranes were photographed and quantified using ChemiDoc XRS+ imaging acquiring system (BioRad) and Image Lab software (BioRad).

### 4.7. TOPflash Assay

Cells were seeded one day before transfection into a 24-well plate in 1 mL cell culture medium. At the time of transfection, cells displayed a confluence of approximately 50%. Cells were transfected with pGL4.49[luc2P/TCF-LEF/Hygro] and FuGENE^®^ transfection reagent following manufacturer’s protocol (Promega, Mannheim, Germany). Two days later, cell medium was changed to selection medium containing 100 μg/mL hygromycin (Invivogen, Toulouse, France). Afterwards, cells were singularized in a 96-well plate, and single clones were picked and then cultivated until confluence was reached. Backups were stored at -80°C until luminescence measurements were performed. Therefore, 1 × 10^4^ cells were seeded per well, in a white 96-well plate, in a total volume of 100 μL. Luminescence intensity was determined using cells cultured on coated collagen surface and/or incubated with manganese, as described under cell culture and reagents. Cells were then incubated for 72 h with the addition of CDDP at a concentration of 2 μM. Luciferase activity was analysed by adding 100 μL ONE-Glo™ Luciferase Assay System to each well (Promega, Mannheim, Germany). After 5 min of lysis, luminescence intensity was detected by using a FLUOstar Optima (BMG Labtech, Ortenberg, Germany). Values were normalized using CellTiter-Fluor^TM^ assay, according to manufacturer’s instructions, for multiplexing (Promega, Mannheim, Germany).

### 4.8. Statistical Analysis

Comparisons were performed using the software Prism™ (GraphPad Software, San Diego, CA, USA). MTT results were analysed by generating sigmoidal concentration-response curves to determine the IC_50_, which was used to calculate the resistance factor (RF), which consists of the ratio of the IC_50_ of treated cells (resistant) by the IC_50_ of control cells (sensitive). Moreover, statistical analysis was performed using one way ANOVA and Dunnett’s test (* *p* < 0.05; ** *p* < 0.01; *** *p* < 0.001).

## 5. Conclusions

The data of this paper introduce a novel functional axis combining integrin activation, by manganese, its specific ligand, most likely via the PI3K/AKT pathway with the wnt signalling pathway as a key phenomenon to explain a lower sensitivity of melanoma cells towards cisplatin cytotoxicity. This expands the present knowledge on the phenomenon of cell adhesion mediated resistance and adds a further functional aspect to it. The two scenarios, which were until now separated, can be interlinked as the general role of integrins in cancer malignancy and the wnt pathway, as a trigger of a bad prognosis come together. A further insight into this connection can help to offer novel therapeutic approaches to interfere with resistance formation. 

## Figures and Tables

**Table 1:** Cisplatin IC_50_ on MV3 cell line.

Treatment	pIC_50_ ± SEM	IC_50_ (μM)	RF
Control	5.00 ± 0.32	10.0	-
Mn^2+^	4.95 ± 0.34	11.3	1.1
Control	4.96 ± 0.18	10.9	-
Collagen	4.84 ± 0.21	14.5	1.2
Control	4.88 ± 0.27	13.1	-
Collagen + Mn^2+^	4.83 ± 0.29	14.9	1.1

Displays the IC_50_ values as well as the pIC_50_, which is the negative log of the IC_50_ used to calculate the standard error of mean (SEM) to obtain a symmetrical fit surrounding the IC_50_. Cells were submitted to MTT assay (3-(4,5-dimethylthiazol-2-yl)-2,5-diphenyltetrazolium bromide), after 72 h of incubation with cisplatin under integrin activating stimuli 1 mM manganese or collagen coating. MTT assay revealed slight but consistent decrease in sensitivity of MV3 melanoma cells after integrin activation. Measurements were conducted in triplicates (at least *n* = 3).

**Table 2:** IC_50_ of cisplatin cytotoxicity on MV3 cells treated with 100 nM BEZ235.

Treatment	pIC_50_ ± SEM	IC_50_ (μM)
Control	4.55 ± 0.33	28.3
BEZ235 100 nM	4.87 ± 0.31	13.4
Mn^2+^ + BEZ235	4.92 ± 0.28	12.0
Collagen + BEZ235	4.83 ± 0.27	14.6
Collagen + Mn^2+^ + BEZ235	4.89 ± 0.31	12.8

## Supplementary Materials

The following are available online at http://www.mdpi.com/2072-6694/9/9/125/s1, Figure S1: BEZ235 cytotoxicity curve. Figure S2: EMT markers. Figure S3: TOPflash assay.
