# Supplementary material for: Integrin Activation Contributes to Lower Cisplatin Sensitivity in MV3 Melanoma Cells by Inducing the Wnt Signalling Pathway
Source: Cancers (Basel). 2017 Sep 16;9(9):125. doi: 10.3390/cancers9090125 (PMC5615340; doi:10.3390/cancers9090125)
Supplement: Supplementary file 1 [file cancers-09-00125-s001.pdf]

Supplementary Materials: Integrin Activation Contributes to Lower Cisplatin Sensitivity in MV3 Melanoma Cells by Inducing the Wnt Signalling Pathway

Maria B. R. Piva, Bastian Jakubzig and Gerd Bendas

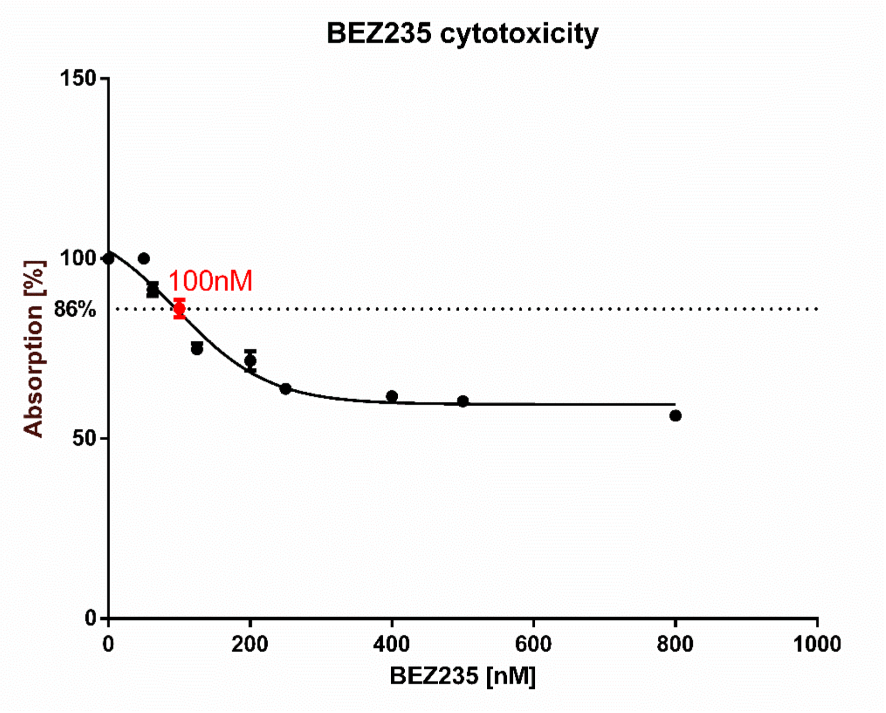

Figure S1. BEZ235 cytotoxicity curve.

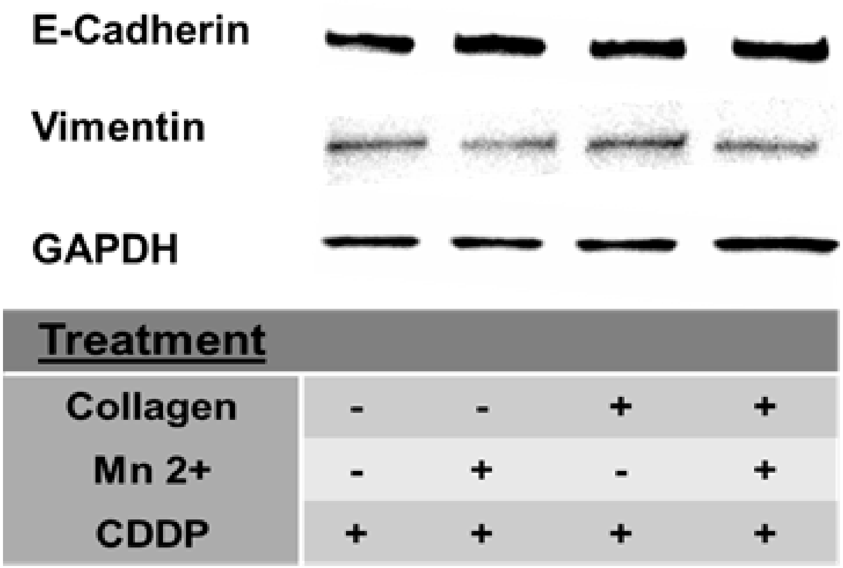

Figure S2. EMT markers.

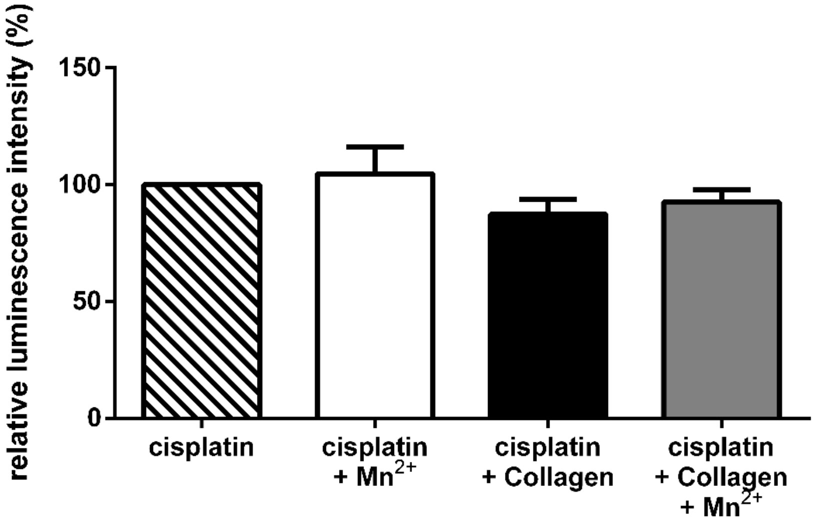

Figure S3. TOPflash assay.

© 2017 by the authors; licensee MDPI, Basel, Switzerland. This article is an open access article distributed under the terms and conditions of the Creative Commons by Attribution (CC-BY) license (http://creativecommons.org/licenses/by/4.0/).
